# Paternal childcare in early childhood and problematic behavior in children: a population-based prospective study in Japan

**DOI:** 10.1186/s12887-021-02838-2

**Published:** 2021-09-10

**Authors:** Manami Ochi, Takeo Fujiwara

**Affiliations:** 1 Department of Health and Welfare Services, National Institute of Public Health, 2-3-6 Minami, Wako City, Saitama, Japan; 2 Department of Global Health Promotion, Tokyo Medical and Dental University, 1-5-45 Yushima, Bunkyo-ku, Tokyo, Japan

**Keywords:** Father, Behavioral problem, Parenting, Early childhood, Longitudinal data

## Abstract

**Background:**

There have been numerous reports on the effects of paternal childcare on children’s behavioral development. However, little is known about these effects in Asian countries such as Japan, where fathers do not have sufficient time for childcare due to long working hours. This study explored the association between paternal childcare during toddlerhood in terms of childcare hours and the type of caregiving behavior and subsequent behavioral problems in children aged 5.5 years, stratified by sex.

**Methods:**

We analyzed data from the Longitudinal Survey of Newborns in the twenty-first Century (2001–2006), a population-based cohort survey in Japan (*N* = 27,870). Paternal childcare was assessed at 18 months in terms of paternal childcare hours on weekdays or weekends and the frequency of each type of childcare (feeding, changing diapers, bathing, putting the child to sleep, playing with the child at home, and taking the child outside). Based on the frequency or lack of paternal involvement, six categories of child behavioral problems were assessed when the children were 5.5 years old. Logistic regression analysis was applied to account for the known confounding variables.

**Results:**

Longer paternal childcare hours, on both weekdays and weekends in toddlerhood, had a protective effect on behavioral problems at 5.5 years of age. The dose-effect relationships were found between the frequency of fathers taking their children outside and behavioral problems in boys, and the frequency of fathers playing with their children at home and behavioral problems in both boys and girls.

**Conclusions:**

Paternal childcare during toddlerhood could prevent subsequent behavioral problems in children. Several specific paternal caregiving behaviors, such as taking their children outside and playing with them at home, may play an important role in preventing subsequent behavioral problems.

**Supplementary Information:**

The online version contains supplementary material available at 10.1186/s12887-021-02838-2.

## Background

An increasing body of research has shown that paternal childcare in early childhood can contribute to child socioemotional development and well-being in various ways [1]. While most studies focus on the importance of mother-baby interaction in childcare, increased involvement in childcare by fathers is associated with multiple aspects of child health and well-being, such as higher cognitive skills [2, 3], receptive language skills [3, 4], better anthropometric outcomes [5], improved social competence [6], higher educational attainment [7, 8], and fewer injuries [9].

Although there is evidence to support the positive impact of fathers’ involvement on children’s social and behavioral outcomes [10], other evidence on the specific benefits of paternal engagement with children during toddlerhood and its impact on children’s behavioral outcome remain limited [11–13]. Direct engagement with children includes caregiving behaviors, such as feeding, changing diapers, playing, and social activities that promote the socio-psychological development of children [1]. Childcare provided by fathers may lead to increased maternal relaxation time and improved maternal mental health and may eventually affect their interactions with their children. Therefore, future research needs to consider not only the duration but also the extent of paternal childcare [14].

Certain types of paternal involvement with children may be partly determined by the child’s characteristics. For example, previous studies using path analysis found bidirectional associations between paternal involvement and child behavior, and a tendency for children with conduct problems or hyperactivity to have less involved fathers at subsequent ages [13, 15]. Children’s characteristics are also strongly associated with their developmental behaviors. Paternal childcare is responsive to the child’s temperament or disabilities, and children with difficulties may elicit less paternal involvement [15, 16]; however, few studies have taken this into account.

Further, previous studies on paternal childcare and its effect on child behavior have been limited to Western countries [17]. Parental behavior is highly context-dependent and diverse depending on various societal factors: geographical conditions, family characteristics, economic status, work-related factors, societal norms and beliefs, and so on [18, 19]. In Japan, where working and commuting hours are longer than in Western countries, many fathers are unable to spend a satisfactory amount of time with their children [20], and the impact of paternal engagement in childcare on children remains unclear.

This study aims to clarify the impact of paternal childcare during toddlerhood on behavioral problems among preschoolers in Japan, in terms of 1) childcare hours and 2) the type of caregiving in the Japanese population.

## Methods

### Study sample

We used data from the Longitudinal Survey of Newborns in the twenty-first Century, a population-based survey conducted by the Ministry of Health, Labor, and Welfare in Japan. The study sample included all babies born in Japan between January 10 and 17 or July 10 and 17, 2001, using birth records from national vital statistics. The baseline survey was mailed to parents when their infants were 6 months old (*n* = 53,575). After the baseline survey, annual surveys were conducted by sending questionnaires to participants by mail. We used data from 2001, 2002, and 2006 survey waves. We included children who lived with both parents and children whose mothers answered the questionnaire to maintain consistency in the assessment of childcare. We excluded responses with missing data for the variables used in the analysis: child problem behavior, paternal involvement in childcare and domestic chores, maternal involvement in childcare and domestic chores, parental education, parental employment, annual household income in 2002, gestational age, weight at birth, and child temperament.

### Exposure: paternal childcare

Paternal childcare was assessed at 18 months of age using the following two measurements: paternal childcare hours on weekdays or weekends, and frequency of each type of caregiving (feeding, changing diapers, bathing, putting the child to sleep, playing with the child at home, and taking the child outside).

Paternal childcare hours were ascertained by asking the following questions: How much time does the father spend with his child on average in 1 day, except for sleeping hours? Respondents answered in respect of both weekdays and weekends by choosing from the following options: none, < 0.5 h, 0.5–0.9 h, 1–1.9 h, 2–3.9 h, 4–5.9 h, and ≥ 6 h. In general, working fathers are expected to spend more time in childcare on weekends than on weekdays; therefore, we re-defined the categories into “less than 0.5 hours,” “0.5 to 1.9 hours,” and “2 or more hours” for weekdays, and “less than 4 hours,” “4 to 5.9 hours,” and “6 or more hours” for weekends.

The frequency of each type of paternal caregiving was retrieved using six items: 1) feeding, 2) changing diapers, 3) bathing, 4) putting the child to sleep, 5) playing with the child at home, and 6) taking the child outside. Responses for each question included “not at all,” “rarely,” “sometimes,” and “always” For these categories, we classified “always” and “sometimes” as a high degree of paternal caregiving, and “rarely” and “not at all” as a low degree of paternal caregiving. To calculate the total paternal caregiving scores, each response was scored from 0 to 3 (i.e., “not at all” = 0 and “always” = 3). The measurement and the method of specifying the caregiving variable were in line with previous studies [9, 21]. We divided the total caregiving scores into three groups, namely the “high degree of paternal caregiving” group with more than 1 SD above the mean, the “low degree of paternal caregiving” group with less than 1 SD under the mean, and the “middle degree of paternal caregiving” group.

### Outcome: child behavioral problems

Child behavioral problems were assessed when the children were 5.5 years old by asking the following six questions that required a yes/no response: 1) Is your child able to listen without fidgeting? 2) Is your child able to focus on a specific task? 3) Is your child patient? 4) Is your child able to express their emotions appropriately? 5) Is your child able to behave in a group situation? 6) Is your child able to keep promises? These variables were developed to identify early signs of behavioral and developmental problems and have been previously used as a set of measurements for behavioral problems [22, 23]. In addition, we defined children with any of these problem behaviors as “children with behavioral problems.”

### Covariates

We selected the following variables as potential confounders: the number of siblings, living with grandparent(s), paternal and maternal age at childbirth (< 25 years, 25–29 years, 30–34 years, 35–39 years, and ≥ 40 years), paternal and maternal education (lower than high school degree, high school degree, 2-year college or vocational school degree, and a college degree or more), annual household income in 2002 (< JPY 4 million, 4–5.9 million, 6–7.9 million, 8–9.9 million, and ≥ 10 million), maternal childcare hours on weekdays or weekends at 18 months old, and total scores of maternal involvement in caregiving. Maternal involvement in caregiving was scored using the same items used to assess fathers’ involvement in caregiving, and the summed score of the six items was used in the analysis.

How fathers are involved with their children is affected by factors relating to the children themselves, such as disabilities and temperament [24–26]. Therefore, the baseline characteristics of children should also be considered when estimating the effect of paternal childcare: gestational age (< 37 weeks, 37–41 weeks, and ≥ 42 weeks), multiple birth (singleton, twins, and triplets), child’s history of hospital admission or visits for congenital diseases, and child’s temperament (self-reported dichotomous answers to questions about 18 traits: active, shy of strangers, playful, short-tempered, careful, aggressive, timid, competitive, obedient, independent, stubborn, full of curiosity, fickle, restless, shy, spoilt, easygoing, and impatient).

### Statistical analysis

We developed logistic regression models for child behavioral problems as predicted by the paternal childcare duration and caregiving score adjusting for covariates shown above. In addition to the crude models, two adjusted models were used: Model 1 adjusted for all covariates shown above, and Model 2 additionally adjusted for other variables of paternal childcare to examine how each of the paternal childcare variables, that is, paternal childcare hours on weekdays, weekends, and total caregiving scores, affected a child’s behavioral problems. We also examined whether the frequency of each type of paternal caregiving was associated with each type of child behavioral problem after adjusting for paternal childcare hours. Finally, to ascertain any evidence of interaction between the main exposure variables and the child’s sex, we conducted a supplementary analysis in which those interaction terms were added into the model.

All analyses were conducted separately for boys and girls because the effects of paternal childcare may differ due to biologically established sex differences in child development [27]. Analyses were performed using Stata version 15.0 (Stata Corp., College Station, TX, USA).

Approval from the ethics committee of the Tokyo Medical and Dental University was waived because the data were anonymous and available from the Ministry of Health, Labour, and Welfare in Japan upon request. Questionnaire responses from caregivers were considered as informed consent to participate in the study.

## Results

A total of 47,015 caregivers responded to the baseline questionnaire in 2001 (response rate: 87.7%), 43,925 (93.4%) caregivers responded in the 2002 survey wave, and 38,540 (82.0%) responded in the 2006 survey wave. We excluded the children who did not live with both parents or whose mothers did not answer the questionnaire (*n* = 3458, 9.0%). We excluded responses that did not comprise the following values: child problem behavior (*n* = 1443, 3.7%), paternal involvement in childcare and domestic chores (*n* = 2533, 6.6%), maternal involvement in childcare and domestic chores (*n* = 1277, 3.3%), parental education (*n* = 578, 1.5%), parental employment (*n* = 807, 2.1%), annual household income in 2002 (*n* = 2705, 7.0%), gestational age (*n* = 14, 0.04%), weight at birth (*n* = 7, 0.02%), and child temperament (*n* = 374, 1.0%). Finally, 27,870 newborns were included in the analyses (72.3% of the respondents in the 2006 survey wave). Paternal caregiving in one type of activity was not strongly correlated with that in another type of activity (Spearman’s correlation coefficients ranging from 0.27–0.50, Table 1). The total paternal caregiving scores ranged from 0 to 18 (Cronbach’s alpha = 0.77).

**Table 1:** Spearman correlation coefficients between specific types of paternal involvement in caregiving

	1)	2)	3)	4)	5)	6)
**1)** Feeding	1.00					
**2)** Changing diaper	0.50	1.00				
**3)** Bathing	0.35	0.31	1.00			
**4)** Putting the child to sleep	0.42	0.42	0.40	1.00		
**5)** Playing with the child at home	0.35	0.31	0.36	0.30	1.00	
**6)** Taking the child outside	0.32	0.30	0.27	0.28	0.44	1.00

Table 2 shows the characteristics of the respondents in total and stratified by child sex. Preterm births and low birth weights accounted for 4.6 and 8.1%, respectively. The mean age of the father and mother at the time of birth was 31.7 (SD = 5.3) and 29.7 (SD = 4.2) years, respectively. Half of the families had older siblings when the participating child was born. One of the five families lived with grandparents. The percentage of the history of hospital visits or admissions due to a child’s congenital diseases was 2.2 and 1.0%, respectively. Large differences in proportion were shown between boys and girls in some of the children’s temperament traits (e.g., 42.8% for playful boys compared to 31.1% for playful girls).

**Table 2:** Characteristics of the participants

	Total		Boy		Girl	
	*n* = 27,870		*n* = 14,429		*n* = 13,441	
	No. or mean	% or SD	No. or mean	% or SD	No. or mean	% or SD
Gestational age (week)						
< 37	1277	4.6	764	5.3	513	3.8
37–41	26,357	94.6	13,542	93.9	12,815	95.3
≥ 42	236	0.8	123	0.9	113	0.8
Birth weight (g)						
≥ 2500	25,616	91.9	13,384	92.8	12,232	91.0
< 2500	2254	8.1	1045	7.2	1209	9.0
Paternal age at child birth (years old)	31.7	5.3	31.8	5.3	31.7	5.3
< 25	1822	6.5	922	6.4	900	6.7
25–29	8060	28.9	4151	28.8	3909	29.1
30–34	10,236	36.7	5332	37.0	4904	36.5
35–39	5583	20.0	2887	20.0	2696	20.1
40+	2105	7.6	1100	7.6	1005	7.5
unknown	64	0.2	37	0.3	27	0.2
Maternal age at child birth (years old)	29.7	4.2	29.7	4.2	29.7	4.2
< 25	2843	10.2	1465	10.2	1378	10.3
25–29	11,059	39.7	5747	39.8	5312	39.5
30–34	10,414	37.4	5402	37.4	5012	37.3
35–39	3204	11.5	1629	11.3	1575	11.7
≥ 40	350	1.3	186	1.3	164	1.2
Paternal education						
less than high school	1936	6.9	1019	7.1	917	6.8
high school	10,773	38.7	5588	38.7	5185	38.6
some college	4461	16.0	2308	16.0	2153	16.0
college or higher	10,700	38.4	5514	38.2	5186	38.6
Maternal education						
less than high school	1076	3.9	539	3.7	537	4.0
high school	10,470	37.6	5414	37.5	5056	37.6
some college	12,074	43.3	6330	43.9	5744	42.7
college or higher	4250	15.2	2146	14.9	2104	15.7
Number of siblings						
none	12,772	45.8	6488	45.0	6284	46.8
1	11,048	39.6	5841	40.5	5207	38.7
≥ 2	4050	14.5	2100	14.6	1950	14.5
Living with grandparent(s) (answer = yes)	5866	21.0	3065	21.2	2801	20.8
Annual household income (JPY million)						
< 4	7966	28.6	4150	28.8	3816	28.4
4–5.9	10,811	38.8	5576	38.6	5235	38.9
6–7.9	5544	19.9	2845	19.7	2699	20.1
8–9.9	2099	7.5	1127	7.8	972	7.2
≥ 10	1450	5.2	731	5.1	719	5.3
Multiple birth						
singleton	27,314	98.0	14,129	97.9	13,185	98.1
twin	546	2.0	295	2.0	251	1.9
triplet	10	0.04	5	0.03	5	0.03
History of hospital visits for child’s congenital diseases (answer = yes)	624	2.2	344	2.4	280	2.1
History of hospital admissions for child’s congenital diseases (answer = yes)	267	1.0	164	1.1	103	0.8
Child’s temperament (answer = yes)						
active	14,737	52.9	7405	51.3	7332	54.5
shy of strangers	2891	10.4	1181	8.2	1710	12.7
playful	10,360	37.2	6182	42.8	4178	31.1
short temper	4551	16.3	2445	16.9	2106	15.7
careful	4172	15.0	2323	16.1	1849	13.8
aggressive	12,169	43.7	5254	36.4	6915	51.4
timid	2084	7.5	1476	10.2	608	4.5
obedient	6513	23.4	3603	25.0	2910	21.7
competitive	8094	29.0	3700	25.6	4394	32.7
independent	12,378	44.4	5465	37.9	6913	51.4
stubborn	3994	14.3	2361	16.4	1633	12.1
full of curiosity	11,305	40.6	5967	41.4	5338	39.7
fickle	4346	15.6	2164	15.0	2182	16.2
restless	6318	22.7	3845	26.6	2473	18.4
shy	9400	33.7	4663	32.3	4737	35.2
spoilt	15,849	56.9	9150	63.4	6699	49.8
easygoing	2142	7.7	1214	8.4	928	6.9
impatient	1622	5.8	845	5.9	777	5.8

*SD* Standard deviation

The distribution of paternal childcare hours and the frequency of each type of caregiving are shown in Table 3. On weekdays, 47.9% of the fathers spent two or more hours with their children. On weekends, 70.1% of the fathers spent six or more hours with their children. Little difference was found in these proportions between boys and girls. The total score of paternal caregiving was distributed normally, with a mean of 11.1 and a standard error of 0.02. For half of the types of caregiving (i.e., bathing, putting the child to sleep, and taking the child outside), the proportion of fathers who were involved in caregiving “sometimes” or “always,” was higher in case of boys than girls.

**Table 3:** The distributions of paternal childcare

	Total *n* = 27,870		Boy *n* = 14,429		Girl *n* = 13,441		*p* value*
	No. or mean	% or SE	No. or mean	% or SE	No. or mean	% or SE	
Childcare hour on weekdays (hour)							
< 0.5	4747	17.0	2456	17	2291	17.0	0.89
0.5–0.9	3654	13.1	1905	13.2	1749	13.0	
1–1.9	6104	21.9	3141	21.8	2963	22.0	
2–3.9	9432	33.8	4864	33.7	4568	34.0	
4–5.9	3348	12.0	1749	12.1	1599	11.9	
≥ 6	585	2.1	314	2.2	271	2.0	
Childcare hour on weekends (hour)							
< 0.5	299	1.1	158	1.1	141	1.0	0.77
0.5–0.9	470	1.7	243	1.7	227	1.7	
1–1.9	1027	3.7	521	3.6	506	3.8	
2–3.9	2521	9.0	1326	9.2	1195	8.9	
4–5.9	4014	14.4	2044	14.2	1970	14.7	
≥ 6	19,539	70.1	10,137	70.3	9402	70.0	
Total caregiving score (range: 0 to 18)	11.1	0.02	11.2	0.03	11.0	0.03	< 0.001
Frequency of feeding							
not at all	3336	12.0	1733	12.0	1603	11.9	0.28
rarely	7346	26.4	3738	25.9	3608	26.8	
sometimes	14,593	52.4	7586	52.6	7007	52.1	
always	2595	9.3	1372	9.5	1223	9.1	
Frequency of changing diaper							
not at all	3416	12.3	1763	12.2	1653	12.3	0.72
rarely	6571	23.6	3396	23.5	3175	23.6	
sometimes	15,631	56.1	8078	56.0	7553	56.2	
always	2252	8.1	1192	8.3	1060	7.9	
Frequency of bathing							
not at all	1337	4.8	612	4.2	725	5.4	< 0.001
rarely	2612	9.4	1291	8.9	1321	9.8	
sometimes	13,954	50.1	7181	49.8	6773	50.4	
always	9967	35.8	5345	37.0	4622	34.4	
Frequency of putting the child to sleep							
not at all	5601	20.1	2703	18.7	2898	21.6	< 0.001
rarely	8268	29.7	4218	29.2	4050	30.1	
sometimes	10,950	39.3	5803	40.2	5147	38.3	
always	3051	10.9	1705	11.8	1346	10.0	
Frequency of playing with the child at home							
not at all	165	0.6	82	0.6	83	0.6	0.61
rarely	1126	4.0	582	4.0	544	4.0	
sometimes	13,846	49.7	7120	49.3	6726	50.0	
always	12,733	45.7	6645	46.1	6088	45.3	
Frequency of taking the child outside							
not at all	917	3.3	457	3.2	460	3.4	< 0.001
rarely	3717	13.3	1826	12.7	1891	14.1	
sometimes	18,882	67.8	9805	68.0	9077	67.5	
always	4354	15.6	2341	16.2	2013	15.0	

*SE* Standard error, * Chi-square test

Table 4 shows the mean of the number of behavioral problems and the percentage of each behavioral problem by sex. Being unable to focus on a specific task was the most observed behavioral problem: 23.9% of the children. Being unable to express emotions appropriately was the second highest (22.9%), followed by being unable to keep promises (18.9%) and being unable to listen without fidgeting (17%). Boys were more likely to have behavioral problems than girls (1.2 vs. 0.8) and had a significantly higher proportion of behavioral problems than girls, except for “being unable to focus on a specific task.”

**Table 4:** The number of children with behavioral problems

	Total *n* = 27,870		Boy *n* = 14,429		Girl *n* = 13,441		*p* value*
	mean or No.	SE or %	mean or No.	SE or %	mean or No.	SE or %	
Number of behavioral problem	1.0	0.01	1.2	0.01	0.8	0.01	< 0.001
Any of behavioral problems	13,995	50.2	7980	55.3	6015	44.8	< 0.001
Unable to listen without fidgeting	4751	17.0	3131	21.7	1620	12.1	< 0.001
Unable to focus on a specific task	3463	12.4	1833	12.7	1630	12.1	0.15
Unable to be patient	6656	23.9	3930	27.2	2726	20.3	< 0.001
Unable to express emotions appropriately	6375	22.9	3672	25.4	2703	20.1	< 0.001
Unable to behave in a group situation	1656	5.9	1086	7.5	570	4.2	< 0.001
Unable to keep promises	5270	18.9	3117	21.6	2153	16.0	< 0.001

*SE* Standard error, * t-test or Chi-square test

Table 5 shows the results of the logistic regression analysis to estimate how paternal childcare contributed to behavioral problems in boys and girls. In Model 1, adjusting for covariates, children whose fathers spent two or more hours on weekdays with them had a smaller number of behavioral problems (for boys: OR: 0.70, 95% CI: 0.60 to 0.82; for girls: OR: 0.81, 95% CI: 0.70 to 0.95) as compared with children whose fathers spent less than half an hour on weekdays with them. In addition, children whose fathers spent six or more hours with them on weekends had fewer behavioral problems (for boys: OR: 0.81, 95% CI: 0.73 to 0.90; for girls: OR: 0.85, 95% CI: 0.76 to 0.94) than children whose fathers spent less than 4 h with them on weekends. Children who received a high degree of paternal caregiving, defined as more than 1 SD above the mean of the total paternal caregiving score (i.e., 14.3 points), were observed to have a smaller number of behavioral problems (for boys: OR: 0.76, 95% CI: 0.67 to 0.88; for girls: OR: 0.77, 95% CI: 0.67 to 0.89) as compared with children who received a low degree of paternal caregiving. When paternal childcare hours and paternal caregiving scores were adjusted simultaneously in Model 2, the association between paternal childcare hours and problem behaviors remained in boys (for weekdays: OR: 0.71, 95% CI: 0.60 to 0.84; for weekends: 0.86, 95% CI: 0.76 to 0.96 in Table 5), but there was no significant association between total caregiving score and problem behaviors in boys. For girls, the association between paternal childcare hours on weekdays and problem behaviors remained (OR: 0.83, 95% CI: 0.71 to 0.99). Paternal childcare hours on weekends were protective against problem behaviors in girls, although the difference was not statistically significant (OR: 0.89, 95% CI: 0.79 to 1.00). There was no significant multicollinearity among the variables adjusted for in Model 2. The coefficients of covariates adjusted in Models 1 and 2 of Table 5 are shown in the Additional Tables 1, 2, 3, and 4.

**Table 5:** The effects of paternal childcare-hour and caregiving score on behavioral problems in 5.5 year-old children; result of the logistic regression model

	Crude model			Model 1			Model 2		
	OR	95%CI		OR	95%CI		OR	95%CI	
**Boys**									
Childcare-hour on weekdays (ref. < 0.5 h)									
0.5 to 1.9 h	0.91	0.78	1.05	**0.82**	**0.70**	**0.96**	0.82	0.70	0.96
≥ 2 h	0.87	0.75	1.01	**0.70**	**0.60**	**0.82**	**0.71**	**0.60**	**0.84**
	(*p* for trend) 0.07			(*p* for trend) **< 0.001**			(*p* for trend) **< 0.001**		
Childcare-hour on weekends (ref. < 4 h)									
4 to 5.9 h	0.93	0.82	1.05	0.92	0.81	1.05	0.96	0.84	1.10
≥ 6 h	**0.79**	**0.72**	**0.87**	**0.81**	**0.73**	**0.90**	**0.86**	**0.76**	**0.96**
	(*p* for trend) **< 0.001**			(*p* for trend) **< 0.001**			(*p* for trend) **0.004**		
Total caregiving score (ref. low)									
middle	0.92	0.83	1.02	**0.87**	**0.78**	**0.97**	0.98	0.87	1.10
high	**0.86**	**0.76**	**0.98**	**0.76**	**0.67**	**0.88**	0.92	0.79	1.07
	(*p* for trend) 0.03			(*p* for trend) **< 0.001**			(*p* for trend) 0.29		
**Girls**									
Childcare-hour on weekdays (ref. < 0.5 h)									
0.5 to 1.9 h	0.94	0.81	1.09	0.90	0.77	1.05	0.91	0.78	1.06
≥ 2 h	0.95	0.82	1.11	**0.81**	**0.70**	**0.95**	**0.83**	**0.71**	**0.99**
	(*p* for trend)			(*p* for trend) **0.001**			(*p* for trend) **0.02**		
Childcare-hour on weekends (ref. < 4 h)									
4 to 5.9 h	0.99	0.88	1.12	1.00	0.88	1.14	1.05	0.91	1.20
≥ 6 h	**0.79**	**0.72**	**0.87**	**0.85**	**0.76**	**0.94**	0.89	0.79	1.00
	(*p* for trend) **< 0.001**			(*p* for trend) **< 0.001**			(*p* for trend) **0.01**		
Total caregiving score (ref. low)									
middle	0.91	0.82	1.00	**0.85**	**0.76**	**0.94**	0.91	0.81	1.01
high	0.90	0.78	1.02	**0.77**	**0.67**	**0.89**	0.88	0.75	1.03
	(*p* for trend) 0.10			(*p* for trend) **< 0.001**			(*p* for trend) 0.11		

^a^Model 1 adjusted covariates variables (number of siblings, living with grandparent(s), paternal and maternal age at child birth, paternal and maternal education, annual household income, maternal childcare-hours on weekdays or weekends at 18 months old, and the total scores of maternal involvement in caregiving, multiple birth, gestational age, child’s history of hospital admission or visits for congenital diseases, child’s temperament).

^b^Model 2 adjusted for Model 1 and other veriables of paternal childcare.

^c^Bold values denote statistical significance at the *p* < 0.05

*OR* Odds ratio; *CI* Confidence interval

Several paternal caregiving behaviors decreased specific problem behaviors in children, as shown in Tables 6 and 7. For example, boys whose fathers always fed them had fewer behavioral problems, such as not listening without fidgeting (OR: 0.74, 95% CI: 0.61 to 0.90), not being patient (OR: 0.76, 95% CI: 0.63 to 0.90), and not keeping promises (OR: 0.82, 95% CI: 0.68 to 0.99, Table 6). In addition, boys whose fathers always changed their diapers had fewer behavioral problems such as not listening without fidgeting (OR: 0.73, 95% CI: 0.60 to 0.90), not focusing on a specific task (OR: 0.74, 95% CI: 0.58 to 0.95), and not expressing their emotions appropriately (OR: 0.80, 95% CI: 0.66 to 0.97). Paternal childcare, such as playing with their children at home or taking their children outside, had a protective dose-effect on most behavioral problems in boys at age 5.5 (all p for trend < 0.05, except for expressing emotions appropriately).

**Table 6:** The effects of paternal childcare on behavioral problems in 5.5 year-old boys; result of the logistic regression model

	Behavioral problem			Unable to listen without fidgeting			Unable to focus on a specific task			Unable to be patient			Unable to express emotions appropriately			Unable to behave in a group situation			Unable to keep promises		
	OR	95%CI		OR	95%CI		OR	95%CI		OR	95%CI		OR	95%CI		OR	95%CI		OR	95%CI	
Frequency of feeding (ref. not at all)																					
rarely	**0.85**	**0.76**	**0.97**	**0.80**	**0.70**	**0.93**	0.99	0.83	1.19	**0.81**	**0.71**	**0.93**	0.88	0.77	1.01	0.89	0.72	1.10	0.93	0.80	1.07
sometimes	**0.86**	**0.77**	**0.97**	**0.86**	**0.75**	**0.98**	1.02	0.86	1.21	**0.82**	**0.72**	**0.93**	0.88	0.78	1.00	0.90	0.74	1.10	0.92	0.81	1.06
always	**0.85**	**0.73**	**0.99**	**0.74**	**0.61**	**0.90**	0.87	0.69	1.11	**0.76**	**0.63**	**0.90**	0.87	0.73	1.04	0.91	0.68	1.21	**0.82**	**0.68**	**0.99**
	(*p* for trend) 0.07			(*p* for trend) **0.04**			(*p* for trend) 0.55			(*p* for trend) **0.004**			(*p* for trend) 0.12			(*p* for trend) 0.51			(*p* for trend) 0.09		
Frequency of changing diaper (ref. not at all)																					
rarely	0.90	0.80	1.02	**0.84**	**0.72**	**0.97**	0.87	0.73	1.04	0.90	0.79	1.03	0.97	0.85	1.11	0.99	0.79	1.24	0.96	0.83	1.11
sometimes	0.94	0.84	1.05	**0.84**	**0.73**	**0.96**	0.95	0.81	1.13	0.96	0.85	1.09	0.90	0.79	1.02	1.07	0.87	1.32	1.03	0.90	1.18
always	**0.82**	**0.70**	**0.97**	**0.73**	**0.60**	**0.90**	**0.74**	**0.58**	**0.95**	0.88	0.74	1.06	**0.80**	**0.66**	**0.97**	1.14	0.84	1.54	0.87	0.71	1.06
	(*p* for trend) 0.14			(*p* for trend) **0.01**			(*p* for trend) 0.27			(*p* for trend) 0.58			(*p* for trend) **0.01**			(*p* for trend) 0.26			(*p* for trend) 0.80		
Frequency of bathing (ref. not at all)																					
rarely	0.92	0.75	1.13	1.18	0.92	1.51	0.89	0.66	1.20	1.17	0.94	1.46	0.81	0.65	1.02	0.93	0.66	1.30	1.03	0.81	1.31
sometimes	1.00	0.83	1.19	1.09	0.88	1.35	0.96	0.74	1.24	1.01	0.83	1.23	0.88	0.73	1.07	0.80	0.59	1.08	1.10	0.89	1.37
always	0.88	0.73	1.07	1.00	0.79	1.24	0.81	0.62	1.05	0.94	0.76	1.15	**0.79**	**0.64**	**0.96**	0.81	0.59	1.11	1.13	0.90	1.40
	(*p* for trend) 0.14			(*p* for trend) 0.17			(*p* for trend) 0.06			(*p* for trend) **0.03**			(*p* for trend) **0.04**			(*p* for trend) 0.16			(*p* for trend) 0.20		
Frequency of putting the child to sleep (ref. not at all)																					
rarely	0.99	0.89	1.10	1.01	0.89	1.14	0.92	0.78	1.07	0.93	0.83	1.04	0.91	0.81	1.02	0.88	0.72	1.06	1.13	1.00	1.28
sometimes	0.97	0.88	1.07	1.06	0.94	1.20	1.05	0.90	1.21	0.93	0.83	1.04	**0.88**	**0.78**	**0.98**	0.92	0.76	1.10	1.08	0.96	1.22
always	1.04	0.91	1.19	1.06	0.90	1.25	0.89	0.72	1.09	0.90	0.77	1.04	0.94	0.80	1.09	1.14	0.90	1.45	1.20	1.02	1.41
	(*p* for trend) 0.93			(*p* for trend) 0.31			(*p* for trend) 0.99			(*p* for trend) 0.20			(*p* for trend) 0.13			(*p* for trend) 0.46			(*p* for trend) 0.11		
Frequency of playing with the child at home (ref. not at all)																					
rarely	1.15	0.70	1.89	1.05	0.60	1.82	0.95	0.48	1.90	0.97	0.58	1.62	0.74	0.44	1.23	0.78	0.39	1.55	0.98	0.56	1.72
sometimes	1.06	0.66	1.70	0.73	0.43	1.23	0.81	0.42	1.57	0.86	0.53	1.40	0.77	0.48	1.25	0.55	0.29	1.06	0.90	0.53	1.53
always	0.95	0.59	1.51	0.64	0.38	1.10	0.75	0.38	1.45	0.79	0.48	1.30	0.65	0.40	1.05	**0.50**	**0.26**	**0.98**	0.82	0.48	1.40
	(*p* for trend) **0.002**			(*p* for trend) **< 0.001**			(*p* for trend) **0.04**			(*p* for trend) **0.02**			(*p* for trend) **< 0.001**			(*p* for trend) **0.01**			(*p* for trend) **0.02**		
Frequency of taking the child outside (ref. not at all)																					
rarely	1.05	0.84	1.31	0.85	0.66	1.09	1.03	0.76	1.41	0.91	0.72	1.15	0.90	0.71	1.15	0.88	0.62	1.25	1.01	0.79	1.29
sometimes	0.92	0.75	1.13	**0.75**	**0.60**	**0.95**	0.86	0.64	1.15	**0.80**	**0.65**	**1.00**	0.89	0.71	1.11	0.76	0.54	1.05	0.86	0.69	1.09
always	0.89	0.71	1.11	0.78	0.60	1.00	0.83	0.61	1.15	**0.78**	**0.62**	**0.99**	0.82	0.64	1.04	0.73	0.51	1.06	0.80	0.62	1.03
	(*p* for trend) **0.02**			(*p* for trend) **0.05**			(*p* for trend) **0.04**			(*p* for trend) **0.01**			(*p* for trend) 0.08			(*p* for trend) **0.05**			(*p* for trend) **0.004**		

^a^Adjusted covariates (number of siblings, living with grandparent(s), paternal and maternal age at child birth, paternal and maternal education, annual household income, maternal childcare hours on weekdays or weekends at 18 months old, and the total scores of maternal involvement in caregiving, gestational age, multiple birth, child’s history of hospital admission or visits for congenital diseases, child’s temperament), and parental spending hours with children.

^b^ Bold values denote statistical significance at the *p* < 0.05

*OR* Odds ratio; *CI* Confidence interval

**Table 7:** The effects of paternal childcare on behavioral problems in 5.5 year-old girls; result of the logistic regression model

	Behavioral problem			Unable to listen without fidgeting			Unable to focus on a specific task			Unable to be patient			Unable to express emotions appropriately			Unable to behave in a group situation			Unable to keep promises		
	OR	95%CI		OR	95%CI		OR	95%CI		OR	95%CI		OR	95%CI		OR	95%CI		OR	95%CI	
Frequency of feeding (ref. not at all)																					
rarely	1.05	0.92	1.18	0.96	0.79	1.16	0.95	0.79	1.15	0.96	0.82	1.11	1.02	0.88	1.18	0.87	0.65	1.15	1.15	0.97	1.36
sometimes	0.94	0.84	1.06	0.96	0.80	1.16	0.86	0.72	1.03	0.87	0.76	1.01	0.96	0.83	1.11	0.89	0.68	1.16	1.04	0.88	1.22
always	1.01	0.85	1.19	0.85	0.66	1.10	0.97	0.76	1.24	**0.74**	**0.60**	**0.91**	1.05	0.86	1.29	1.10	0.75	1.61	1.15	0.92	1.44
	(*p* for trend) 0.23			(*p* for trend) 0.38			(*p* for trend) 0.22			(*p* for trend) **0.002**			(p for trend) 0.78			(p for trend) 0.92			(p for trend) 0.86		
Frequency of changing diaper (ref. not at all)																					
rarely	0.99	0.88	1.13	0.95	0.78	1.15	0.91	0.76	1.11	0.87	0.75	1.01	1.06	0.91	1.24	1.12	0.84	1.50	1.05	0.89	1.24
sometimes	0.93	0.83	1.05	0.90	0.76	1.08	0.95	0.80	1.14	**0.85**	**0.74**	**0.98**	0.97	0.84	1.13	1.00	0.76	1.32	0.99	0.85	1.16
always	0.97	0.81	1.15	0.88	0.68	1.14	0.97	0.75	1.25	**0.74**	**0.60**	**0.92**	1.13	0.92	1.40	1.08	0.71	1.64	1.00	0.80	1.25
	(*p* for trend) 0.21			(*p* for trend) 0.22			(*p* for trend) 0.92			(*p* for trend) **0.01**			(*p* for trend) 0.97			(*p* for trend) 0.83			(*p* for trend) 0.73		
Frequency of bathing (ref. not at all)																					
rarely	0.95	0.79	1.15	1.11	0.84	1.48	1.03	0.78	1.36	0.91	0.73	1.15	0.95	0.76	1.19	1.09	0.72	1.63	0.84	0.66	1.08
sometimes	0.91	0.77	1.07	0.86	0.67	1.10	0.87	0.68	1.11	0.92	0.76	1.12	0.93	0.77	1.13	0.82	0.57	1.17	0.82	0.66	1.01
always	0.88	0.74	1.05	0.82	0.63	1.07	0.88	0.69	1.15	0.92	0.75	1.14	0.92	0.75	1.14	0.76	0.52	1.12	0.93	0.75	1.16
	(*p* for trend) 0.13			(*p* for trend) **0.01**			(*p* for trend) 0.19			(*p* for trend) 0.67			(*p* for trend) 0.46			(*p* for trend) **0.03**			(*p* for trend) 0.67		
Frequency of putting the child to sleep (ref. not at all)																					
rarely	1.02	0.92	1.13	1.01	0.86	1.19	0.93	0.80	1.08	0.97	0.86	1.10	1.09	0.97	1.24	1.02	0.80	1.29	0.99	0.86	1.13
sometimes	1.03	0.93	1.14	**1.20**	**1.03**	**1.40**	0.87	0.75	1.02	0.93	0.82	1.05	**1.14**	**1.01**	**1.29**	1.05	0.83	1.33	0.95	0.83	1.09
always	0.96	0.83	1.11	1.04	0.83	1.30	0.88	0.71	1.10	0.90	0.76	1.08	1.07	0.90	1.28	0.86	0.60	1.24	0.89	0.73	1.08
	(*p* for trend) 0.94			(*p* for trend) 0.08			(*p* for trend) 0.11			(*p* for trend) 0.16			(*p* for trend) 0.14			(*p* for trend) 0.78			(*p* for trend) 0.21		
Frequency of playing with the child at home (ref. not at all)																					
rarely	0.88	0.54	1.43	0.95	0.48	1.89	0.86	0.44	1.65	0.63	0.38	1.06	1.27	0.70	2.32	0.89	0.36	2.23	0.97	0.52	1.81
sometimes	0.86	0.54	1.35	0.75	0.39	1.44	0.72	0.39	1.34	**0.54**	**0.33**	**0.88**	1.10	0.62	1.95	0.66	0.28	1.56	0.97	0.54	1.75
always	0.75	0.47	1.20	0.64	0.33	1.25	0.67	0.36	1.25	**0.48**	**0.29**	**0.78**	0.96	0.54	1.70	0.56	0.23	1.36	0.83	0.46	1.50
	(*p* for trend) **0.002**			(*p* for trend) **0.001**			(*p* for trend) **0.05**			(*p* for trend) **< 0.001**			(*p* for trend) **0.003**			(*p* for trend) **0.02**			(*p* for trend) **0.01**		
Frequency of taking the child outside (ref. not at all)																					
rarely	**0.78**	**0.63**	**0.97**	0.85	0.62	1.17	0.99	0.71	1.36	0.85	0.66	1.09	0.90	0.70	1.15	0.76	0.49	1.17	0.96	0.73	1.25
sometimes	**0.75**	**0.62**	**0.92**	0.82	0.61	1.11	0.95	0.70	1.29	0.89	0.70	1.13	0.83	0.65	1.05	0.69	0.46	1.04	0.80	0.62	1.03
always	**0.71**	**0.57**	**0.89**	0.79	0.57	1.11	0.90	0.64	1.26	0.82	0.63	1.07	0.77	0.59	1.00	0.73	0.46	1.16	**0.67**	**0.51**	**0.90**
	(*p* for trend) **0.01**			(*p* for trend) 0.24			(*p* for trend) 0.38			(*p* for trend) 0.39			(*p* for trend) **0.03**			(*p* for trend) 0.24			(*p* for trend) **< 0.001**		

^a^Adjusted covariates (number of siblings, living with grandparent(s), paternal and maternal age at child birth, paternal and maternal education, annual household income, maternal childcare hours on weekdays or weekends at 18 months old, and the total scores of maternal involvement in caregiving, gestational age, multiple birth, child’s history of hospital admission or visits for congenital diseases, child’s temperament), and parental spending hours with children.

^b^Bold values denote statistical significance at the *p* < 0.05

*OR* Odds ratio; *CI* Confidence interval

Girls whose fathers always fed them or changed their diapers had fewer problems with patience (OR: 0.74, 95% CI: 0.60 to 0.92) (Table 7). Fathers playing at home with their children had a protective dose-effect on all types of problem behaviors in girls at age 5 (all p for trend < 0.05). Interestingly, girls whose fathers sometimes put them to sleep had more problematic behaviors, such as always fidgeting while listening (OR: 1.20, 95% CI: 1.03 to 1.40) and not expressing their emotions appropriately (OR: 1.14, 95% CI: 1.01 to 1.29) than girls whose fathers never put them to sleep.

Additional Tables 5 and 6 show the results of adding to the model the interaction terms between each main exposure and sex. Most results confirm the main effect that girls have less problematic behavior than boys, although the effects of interaction were not clear.

## Discussion

The present study examined the association between paternal childcare during toddlerhood and behavioral problems at 5.5 years of age using a Japanese nationwide population-based longitudinal cohort. Our findings suggest that longer paternal childcare hours in the toddler period were associated with a lower risk of behavioral problems at 5.5 years of age, even after adjusting for paternal parenting behaviors in toddlerhood. We also found that several paternal caregiving behaviors showed a strong preventive effect on specific problem behaviors in children, such as taking the child outside for boys and playing at home for both boys and girls.

The protective effect of paternal involvement in childcare on children’s subsequent behavioral problems is consistent with previous studies that reported that child appropriate behavior was positively associated with parenting by the father, after adjusting for other confounders, including parenting by the mother [10, 12, 28, 29]. The current study also revealed the effect of paternal time spent with children on respective weekdays and weekends on preschooler’s behavior. Previous studies have suggested that paternal behavior is likely to be influenced by the characteristics of employment and workplace [30, 31]. Therefore, these studies noted the importance of separately considering the fathers’ weekday and weekend involvement with their children. However, research thus far has not examined the extent to which childcare on weekdays and weekends predict child behavioral outcomes. The results have important policy implications for populations in Japan, where fathers work long hours, even when their children are infants/toddlers, and thus do not have sufficient time for childcare. On the other hand, the overall frequency of paternal childcare during toddlerhood was not independently associated with behavioral problems in children at 5.5 years of age, after adjusting for the amount of time fathers spent with their children, suggesting that the duration matters for children’s subsequent behavioral problems. Previous studies have reported that fathers’ parenting quality, as well as the quantity of routine care provided, are associated with a lower risk of child behavioral problems [12, 28]. In addition, fathers who spend more time on child caregiving have a higher quality of interaction with their children, because primary caregiving fathers have a better understanding of how to care for and play with their children as a result of the greater time they have spent with them [32]. This study did not directly measure the quality of parenting; however, spending more time with children on weekdays and weekends could improve paternal caregiving skills, which may have a positive impact on children’s behavior.

Previous studies have consistently reported that mothers typically spend a significant number of hours caring for their children, whereas fathers spend a greater number of hours playing with their children. Our findings highlight that the impact of fathers’ involvement on their children’s behavior can differ depending on what type of parenting fathers engage in, such as essential routine cares or play with the child. Children whose fathers were always involved in some type of essential childcare, such as feeding and changing diapers, had fewer problems in specific behaviors. Possibly, paternal childcare, which is essential for a child’s life, might help reduce the mothers’ childcare burdens and contribute to lower maternal stress, which is considered as a risk for behavioral problems in children [33, 34]. On the other hand, playing with children at home or taking them outside is a more complex type of interaction between fathers and children, which is an important stimulus for the child’s socioemotional development [35]. Therefore, in this study, we found that these types of paternal caregiving could have preventive effects on a variety of problem behaviors in children.

We estimated the sex-specific effects of paternal childcare on behavioral problems in children. Our results did not confirm the sex difference in paternal time spent with the child on weekdays and weekends. Fathers of boys, however, were involved slightly more frequently than those of girls in several types of caregiving, such as bathing, putting their children to sleep, and taking them outside. These sex differences in paternal involvement have been found in previous studies and are considered to be induced in response to underlying biological or psychosocial differences in children [27, 36]. Moreover, the influence of paternal caregiving on the child’s behavior differed depending on the child’s sex in this study. For example, boys showed reduced behavioral problems when their fathers often took them outside, while girls showed reduced behavioral problems when their fathers often played with them at home. The reason for these sex-specific effects might be explained by the well-established gender differences in behavior [36–38]; however, further research is required.

This study has several strengths. First, in our analysis, we considered the child’s original temperament and congenital disabilities, which could be related to the involvement of fathers in parenting as well as behavioral problems in their children. Therefore, the present study provides robust evidence that paternal childcare has a positive effect on children’s behavioral problems. Second, these results were obtained from a representative sample in Japan, in which fathers typically spend long hours commuting and working and, therefore, spend less time with their children than fathers in Western countries [39, 40]. This study provided a test of the generalizability of the hypothesis that paternal childcare would have a protective effect on child behavior. Third, we used a prospective cohort study; thus, reverse causation was unlikely.

Despite its strengths, this study also has some limitations. First, we used maternal reports to evaluate paternal childcare because of the lack of precise measures. In addition, our survey did not ask about the actual amount of time spent on each type of paternal caregiving behavior. According to previous studies, mothers’ evaluations of paternal childcare are susceptible to influence by marital satisfaction and maternal emotional well-being [41, 42]. Other objective measures of paternal childcare should be used in future studies. Second, the outcome in this study, that is, behavioral problems in 5.5-year-old children, were also self-reported by their mothers. Mothers may be one of the primary caregivers for children in most cases; however, they may not be able to fully observe their child’s behavior in situations other than at home. Furthermore, the measurements for child problem behavior in this study are not sufficiently validated although they are used in previous studies [22, 23]. However, the behavioral questions in this study are similar to those on the Strengths and Difficulties Questionnaire (SDQ), which is a validated child behavioral screening instrument. The SDQ consists of 25 questions with five subscales: emotional problems, conduct problems, hyperactivity/inattention, peer problems, and prosocial behavior [43, 44]. For example, “unable to focus on a specific task” in our questionnaire is similar to “Restless, overactive, cannot stay still for long” on SDQ, and “behave in a group situation” in our questionnaire is similar to “Shares readily with other children (treats, toys, and pencils, etc.)” on SDQ. Third, our results could still be biased by residual confounding that we could not measure in this survey, such as marital relationships or paternal mental health. Data about the father’s health or relationships with their partners are scarce, so their potential effects on paternal childcare and child development are not widely recognized [45]. Researchers and practitioners would need to take these family factors into account when considering the interactions between fathers and children.

## Conclusions

Despite these limitations, the present study suggests that paternal childcare in the toddlerhood could prevent subsequent behavioral problems in children based on a national prospective study in Japan. Our study also showed that several specific caregiving behaviors by fathers, such as playing with their children at home or taking them outside, may play an important role in appropriate behavioral development in early childhood, which depends on the child’s sex. Promoting paternal childcare support would augment the quantity and quality of paternal caregiving, which in turn could have a beneficial effect on child behavioral development. The policies that support child-rearing fathers, including the promotion of work environments that facilitate paid parental leave, restriction of overtime work, and incentives for remote or flex-time work, could be linked to the prevention of behavioral problems in children. Further studies using more detailed data on paternal childcare are required to elucidate the mechanisms by which fathers’ involvement in childcare could have a protective effect on early childhood behavior.

## Supplementary Information

**Additional file 1: Table S1**.

**Additional file 2: Table S2**.

**Additional file 3: Table S3**.

**Additional file 4: Table S4**.

**Additional file 5: Table S5**.

**Additional file 6: Table S6**.

## Data Availability

The data that support the findings of this study are available from the Ministry of Health, Labor, and Welfare in Japan (MHLW) but restrictions apply to the availability of these data, which were used under license for the current study, and so are not publicly available. The point of contact in MHLW from the data can be requested: Examination and Analysis Office, Director-General for Statistics and Information Policy. Central Joint Government Building No.5, 1–2-2 Kasumigaseki, Chiyoda-ku, Tokyo. 100–8916. Tel: + 81-3-5253-1111 (ext. 7391), Email: mokutekigai@mhlw.go.jp

## Abbreviations

OR: Odds ratio
CI: Confidence interval
SD: Standard deviation
SE: Standard error
